# Science and Scientific Advice in a Time of Crisis

**DOI:** 10.1093/function/zqaa025

**Published:** 2020-10-05

**Authors:** Ole H Petersen

**Affiliations:** School of Biosciences, Cardiff University, Wales, UK

On March 11, 2020, as I boarded my flight back to the UK from a conference in Washington, DC, the World Health Organization (WHO) for the first time described the coronavirus situation as a pandemic. At that point, there was already a substantial amount of valuable information about the new virus. The genetic sequence of the virus, first determined by Chinese scientists, had been shared worldwide already on January 12 and data from Wuhan, where the virus first emerged at the end of 2019, had demonstrated the amazingly rapid spread of the virus, but also the remarkable effectiveness of stopping transmission by a severe and well-controlled lockdown. Since then ∼1 million people have died of COVID-19 worldwide and major parts of the world have suffered and continue to suffer from various forms of lockdown with large sections of society now working from home. Video conferencing has replaced physical committee meetings in most organizations and webinars have replaced scientific conferences. The impact has been enormous, global and, at the time of writing this editorial, the crisis is certainly not yet over.

Inevitably, in a crisis caused by an infectious agent, society at large expects solutions to come from the biomedical scientific establishment and therefore we have seen much debate in the public domain about scientific advice for policy. In the middle of September, I had the privilege of chairing a webinar, organized by Science Advice for Policy by European Academies (SAPEA), on “Science Advice: What Works in a Crisis,” with some of Europe’s most influential science advisors.^1^ At this intermediate point in the crisis, there were large variations in the outcome in different European countries. The UK, for example, had recorded a number of deaths per million population due to COVID-19 that was more than 5 times higher than that in Germany. The UK mortality rate was of similar magnitude, although higher, than those of France, Italy, and Spain, and very close to that of the USA. Germany’s much better result looked comparatively impressive, but was still much worse than that seen in many Asian countries including China. To what an extent different science advice mechanisms in different countries had influenced the outcome was a major focus of the debate, but the issue was complicated because inevitably science advisors had to operate differently in different countries depending on the nature of government and the overall political climate. When, at the end of the webinar, I asked Ortwin Renn (Chair of the Working Group that produced the SAPEA Evidence Review Report on “Making Sense of Science under Conditions of Complexity and Uncertainty”^2^) to briefly state his overall conclusions, he made three points, based on his experience in a democratic country: “Secrecy doesn’t pay off. Hidden agendas backfire. Bringing people into the policymaking process is the best way to get messages across”.^1^ It may be significant, with regard to the outcomes so far, that many countries do not seem to have dealt with the crisis in line with these principles.

Science advise to policymakers should ideally be based on reliable scientific evidence. However, as noted in SAPEA’s MASOS report,^2^ areas of great public concern, when policy decisions are urgently needed, are often areas of considerable complexity in which the evidence is incomplete and disputed. Furthermore, the available remedial actions often carry considerable costs and can have many unintended consequences. COVID-19 is such a case. The only effective remedy known initially, namely the almost total lockdown imposed early and effectively in Wuhan, could not be repeated in, for example, London or New York, not only for political reasons but also because the administrative and technical structures for control and feeding a locked-down population did not exist. Nevertheless, the partial lockdowns that were imposed in various places, although often too late to have maximal effects, still proved at least partially effective.

Since physical distancing between people can never be perfectly achieved, facemasks could be helpful by limiting transmission of the virus. However, in the early part of the crisis, many national scientific advisors, as well as the WHO, took a narrow view and just emphasized that there was no scientific evidence showing that facemasks would be effective and therefore did not recommend their use. In my opinion, they forgot that “Absence of Evidence is not the same as Evidence of Absence.” In other words, the fact that there was no hard evidence showing that facemasks were effective did not mean that there was evidence that they were ineffective. Furthermore, the important precautionary principle would demand that facemasks should be worn, as it is obvious that they would provide a potential barrier to transmission, while having no significant negative effects. Those warning against the general use of facemasks, including the WHO, also employed the strange argument that they could make people feel overconfident and therefore less careful. However, if we were to accept this kind of argument, we should never introduce any measures that could help solve a problem and wearing safety belts in cars, for example, should not have been recommended and certainly never have been made mandatory. As a member of the German National Academy of Sciences Leopoldina, I was happy that this academy, in its public advice published on April 3, 2020 clearly and unambiguously recommended the use of facemasks.^3^ Unfortunately, the significant delay in many countries to recommending and demanding the use of facemasks, which are now generally accepted to be an important tool to combat transmission, undoubtedly cost many lives.

There is often a tendency to think that only those measures that cost a lot of money and require development by large teams are likely to be really effective in solving a particular problem. With regard to COVID-19, massive resources have been invested in developing vaccines, and we must of course hope that they will be effective or at least partially effective. In contrast, there has been comparatively little interest in investigating potential effects of simpler and cheaper interventions. As Editor of *Function*, I was happy to be able to publish, in our very first issue, an Evidence Review by the eminent lipid biochemist Valerie O’Donnell that examined the possibility that oral rinses with certain mouthwashes, available without prescription, could provide an effective defense against COVID-19.^4^ The idea was based on the knowledge that SARS-CoV-2 is an enveloped virus and dissolving its lipid envelope should disable it. Furthermore, viral loads are high in the oral cavity and throat. Gargling with agents that could dissolve the envelope of the virus might therefore be effective.^4^ When the evidence review was first published (5 June), there was still no direct evidence available showing that SARS-CoV-2, specifically, could be disabled in this way. However, on 29 July an original article appeared,^5^ citing the evidence review published in *Function*,^4^ that examined the virus killing activity of mouthwashes, under conditions mimicking the composition of nasopharyngeal secretions. The authors of this study reported that three readily available mouthwashes could rapidly reduce viral infectivity to undetectable levels.^5^ This was *in vitro*, but at least two clinical trials have now been planned to test whether this treatment would work in practice. Unfortunately, it will take time to provide the clinical evidence that may be needed before most scientific advisors would be willing to recommend this cheap and safe procedure. In this case, it is not entirely straightforward to design appropriate clinical trials. Whereas it is relatively easy in test tube experiments to determine precisely the concentration of an agent required to inactivate the virus and the time it takes to accomplish this, it is not so easy to extrapolate from this well-defined situation to the much more complex scenario of treating the human mouth and throat. For how long, and exactly how should one gargle with mouthwash and how many times a day should this be repeated? The question about the required frequency of rinsing is particularly tricky because virus-shedding occurs constantly. “Trial and error” will inevitably be needed and it may well take a considerable amount of time before we know exactly what would be the optimal procedure. However, if information about what is already known were to be shared widely, many individuals might already now decide to gargle a couple of times every day with a mouthwash that has been proven to be able to kill SARS-CoV-2. This might well provide significant protection without doing any harm.

Throughout this crisis, I have often worried about the low level of relevant and practical information that is transmitted to the general population. Even the so-called quality newspapers, for example, seem more interested in reporting “outrageous” comments and disagreements between different factions, than giving specific information that could help people decide on the best measures to protect themselves. It is worrying that the important issue of how best to deal with a disease caused by a well-defined biological agent, in many places has degenerated into slanging matches between highly polarized political groupings. While certain figures in political leadership positions have played and continue to play destructive roles, it is also the case that the news media have not, on the whole, behaved as responsibly as they could. In both the UK and the USA, for example, the media constantly focus on comments concerning the pandemic made by the President or the Prime Minister, as if the opinion of these individuals, who have no background in medicine or science, should carry special weight. These opinions, that often change very quickly, have mostly served to confuse the public and have therefore provided an unhelpful diversion from more valuable information provided by those who possess insight and common sense, based on real knowledge and experience.

As scientists, science advisors, members of academies, and scientific societies, we must all continue to do what we can to counter the infantilizing effect of the news industry, but it will unfortunately continue to be an unequal battle.

## Funding

The author acknowledges funding from the European Commission's Horizon 2020, grant agreement 737432.

## Conflict of interest Statement

None declared.
